# Topological nature and the multiple Dirac cones hidden in Bismuth high-Tc superconductors

**DOI:** 10.1038/srep10435

**Published:** 2015-05-27

**Authors:** Gang Li, Binghai Yan, Ronny Thomale, Werner Hanke

**Affiliations:** 1 Institut für Theoretische Physik und Astrophysik, Universität Würzburg, 97074 Würzburg, Germany; 2 Max Planck Institute for Chemical Physics of Solids, 01187 Dresden, Germany; 3 Max Planck Institute for the Physics of Complex Systems, 01187 Dresden, Germany

## Abstract

Recent theoretical studies employing density-functional theory have predicted BaBiO_3_ (when doped with electrons) and YBiO_3_ to become a topological insulator (TI) with a large topological gap (~0.7 eV). This, together with the natural stability against surface oxidation, makes the Bismuth-Oxide family of special interest for possible applications in quantum information and spintronics. The central question, we study here, is whether the hole-doped Bismuth Oxides, i.e. Ba_1-x_K_x_BiO_3_ and BaPb_1-x_Bi_x_O_3_, which are “high-Tc” bulk superconducting near 30 K, additionally display in the further vicinity of their Fermi energy E_F_ a topological gap with a Dirac-type of topological surface state. Our electronic structure calculations predict the K-doped family to emerge as a TI, with a topological gap above E_F_. Thus, these compounds can become superconductors with hole-doping and potential TIs with additional electron doping. Furthermore, we predict the Bismuth-Oxide family to contain an additional Dirac cone below E_F_ for further hole doping, which manifests these systems to be candidates for both electron- and hole-doped topological insulators.

Topological insulators (TIs) are new quantum states of matter that are of fundamental interest for both condensed-matter physics studies and applications in spintronics, quantum information as well as thermoelectrics^3^,^4^. The unique feature of the TIs is the existence of topologically protected and, therefore, robust conducting channels at the surfaces/edges of materials that are insulating in the interior. Due to the existence of band-inversion in their bulk electronic structure, this new type of insulators is topologically different from the conventional insulators in the sense that TIs cannot be adiabatically transformed into an atomic insulator without going through a phase transition. More precisely, charge conservation and time reversal symmetry around the Fermi level of band inversion establish the protecting symmetry of the non-trivial odd Dirac cone surface state which cannot be gapped out continuously.

Recently, a new direction in the search for topological insulators, with a substantial potential for the above applications, has emerged by identifying BaBiO_3_ as a TI in the electron-doped region^1^,^2^. According to density-functional electronic structure calculations^1^, this compound possesses the largest topological gap (~0.7 *eV*) among currently known TI materials and is naturally stable against surface oxidation and degradation, in contrast to other TIs. The large topological gap is induced by the strong spin-orbit coupling (SOC) of Bismuth in cubic BaBiO_3_, which causes an inversion between the Bi-*s* and Bi-*p* band at a time-reversal invariant momenta (TRIM), *i.e.* the symmetry point R. Inside the corresponding topological gap a Dirac-type of topological surface state (TSS) then exists. So far, however, BaBiO_3_ has not yet experimentally been verified as a topological insulator.

The central question, which we want to address in this work, is to study to what extent the decisive role of the SOC of the *s*- and *p*-Bismuth orbitals for the band inversion and the TI nature can be carried over to the large family of superconducting, *i.e.* doped, Bismuth Oxides. Here of particular interest are the potassium (K) and lead (Pb)-doped “relatives” of BaBiO_3_. As the most experimentally studied doped BaBiO_3_ compounds, these systems are naturally the first choice to understand the influence of doping on the topological nature of BaBiO_3._ It has been known for a long time, that doping cubic BaBiO_3_ with K and Pb will convert this system to a superconductor^5^,^6^(SC). Indeed, Ba_1-x_K_x_BiO_3_ and BaPb_1-x_Bi_x_O_3_ show the highest transition temperatures in copper- and iron-free systems. Thus, the key question here is, whether the doping of BaBiO_3_ with K and Pb, *i.e.* extending the systems to the hole-doped high-Tc bulk superconductors, will still preserve a “hidden” topological insulator phase achievable through *additional* electron- or also hole-doping. Our work answers this crucial question: for the experimentally known structures and phases of the K- and Pb-doped BaBiO_3_ compounds, the topological nature is indeed found to be robust, providing the feasibility of doping, which still keeping the topological nontrivial band structure. This should encourage experimentalists to tune BaBiO_3_ by first achieving an appropriate doping and then performing electric gating on top of this.

These questions are answered via a theoretical study of the other two end compounds, *i.e.* KBiO_3_ and BaPbO_3_, in their cubic phase. Additionally, we consider the doped compounds Ba_0.5_K_0.5_BiO_3_, as well as BaPb_0.7_Bi_0.3_O_3_, which facilitates an interpolation in our search for 3D TIs within the Ba_1-x_K_x_BiO_3_ and BaPb_1-x_Bi_x_O_3_ families.

We find that cubic KBiO_3_ has a very similar electronic structure as BaBiO_3_, including again the band inversions at the R-point. However, compared to the band inversion in BaBiO_3_, this happens at a higher energy in KBiO_3_. Thus, cubic KBiO_3_ can also become a TI via electron doping, albeit experimentally this may harder to be realized. In BaPbO_3_, the Bi *s*-band moves to higher energy and while a direct energy gap is left at the R-point, preserving the topological nature^1^, the indirect gap is zero. As a result, in the surface BZ the Dirac cone merges into the bulk bands. Under electron doping, BaPbO_3_ can then be tuned into a “topological metal”. As an important additional observation, we find that all three Bismuth Oxides, *i.e.* BaBiO_3_, KBiO_3_ and BaPbO_3_ are found to contain another band inversion at the Γ-point *below* the Fermi level. For this reason, these systems can, in principle, also be tuned into TIs via hole doping. These results concerning the topological nature and the “hidden” Dirac cones are also carried over to arbitrary doping levels, as discussed in our work.

## Results

**Figure 1:**
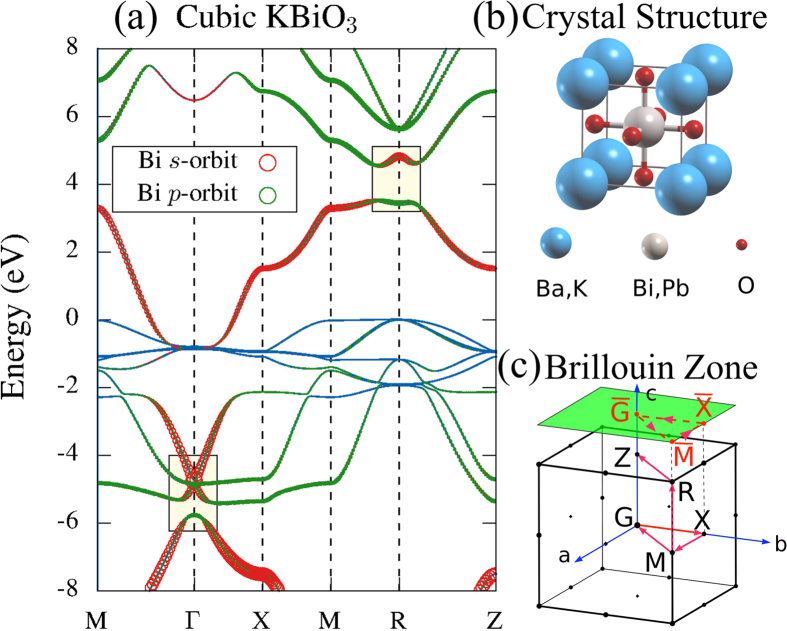
Electronic structure of KBiO_3_: (a) along high-symmetry paths in the first BZ, as indicated by the red arrows in (c) for the crystal structure (b). The width of the red and green colored bands shows the weight of Bi *s*- and *p*-orbitals, the two light-yellow areas mark the band-inversions of this system.

Let us first briefly review the structural aspects of all three parent compounds and explain why we will focus in this paper on their cubic phase. One striking feature in BaBiO_3_ is the breathing-mode distortions, induced by Bi ions. It gives rise to an ordered arrangement of Bi^3+^ and Bi^5+^ ions^7^. Comprehensive crystallographic studies, by using neutron powder diffraction^8^,^9^, found that BaBiO_3_ experiences a number of temperature-induced phase transitions. They range from monoclinic in P2_1_/n at low temperature and monoclinic in I2/m at room temperature to rhombohedral in R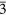 at *ca* 405 K and cubic in Fm
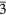
m at *ca* 750-800 K. In ref.^1^, the interesting topological phase was found to exist in the perovskite lattice (see crystal structure in Fig. 1(b)) of the parent compound BaBiO_3_ in the cubic phase, which is stable against the monoclinic lattice distortion. The perovskite structure of BaPbO_3_ is also quite stable^10^,^11^. At temperatures above 673 K, it crystallizes in the simple perovskite structure in Pm
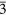
m and transforms to tetragonal I4/mcm structure at temperatures below it. Further decreasing the temperature to 573 K, it changes to the orthorhombic Ibmm structure. KBiO_3_ does not form the perovskite structure but rather crystallizes in a cubic KSbO_3_-type tunnel structure with space group Im
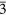
12.

Despite the large variations in their crystal structures, as far as *superconductivity* is concerned, the Ba_1-x_K_x_BiO_3_ compounds are found to confine to their cubic symmetry^13^,^14^,^15^. For this reason, we restrict our study of the parent compound KBiO_3_ to the simple Pm
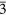
m perovskite structure (see Fig. 1).

The strategy here is as follows: if the two end parent compounds are both TIs in the cubic structure, their random alloys with the same structure would have a large chance to be also TIs. Through first-principle calculations of a superstructure, we confirm this indeed to be the case for Ba_1-x_K_x_BiO_3_. Superconducting BaPb_1-x_Bi_x_O_3_ is confined to the tetragonal distortion of the cubic phase^16^. Similarly to Ba_1-x_K_x_BiO_3_, here we will also concentrate on the cubic phase of BaPbO_3_ and BaPb_1-x_Bi_x_O_3_. In addition, we also show that the tetragonal distortion does not change the topological nature of the cubic BaPbO_3_.

### KBiO_3_ and Ba_0.5_K_0.5_BiO_3_

Figure 1 displays the electronic structure of simple cubic KBiO_3_ with the lattice constant 4.2886 Å^13^,^17^. Its band structure is very similar to that of BaBiO_3_. Thus, the discussions here also apply to BaBiO_3_. Cubic KBiO_3_ is a metal, with a band carrying a large weight of the Bi *s*-orbital crossing the Fermi level. The potassium states stay at higher binding energy and are not relevant to the band-inversions we discuss here. The bands displayed in Fig. 1(a) are mainly from the bismuth and oxygen states. The band-inversions of KBiO_3_ are shown in Fig. 1(a) as the interchange of the red and green colors at special TRIM (see, for example, the light-yellow area around the R-point). The red and green colors are used to label Bi *s*- and *p*-orbitals, respectively. The interchange of the two colors inside the light-yellow area indicates that the order of these two bands is inverted. Due to this reason, the adiabatic connection to the atomic limit of the system is then lost, which makes this system topologically different from the conventional insulator. An important point for the more general TI physics in these compounds is that, the topological gap shown in the light-yellow area at the R-point is induced by the strong SOC of Bi, as discussed in ref.^1^ for BaBiO_3_.

In addition, we found a band inversion at the Γ-point, however, *below* the Fermi level. Similar to the band inversion at R, the Bi *s*-orbital and *p*-orbital weights interchange at Γ around 5.8 *eV* below E_F_. It may experimentally not be easy to shift E_F_ into such heavily hole-doped region. However, it should be feasible for ARPES to detect the corresponding Dirac-type surface states in the occupied bands.

**Figure 2:**
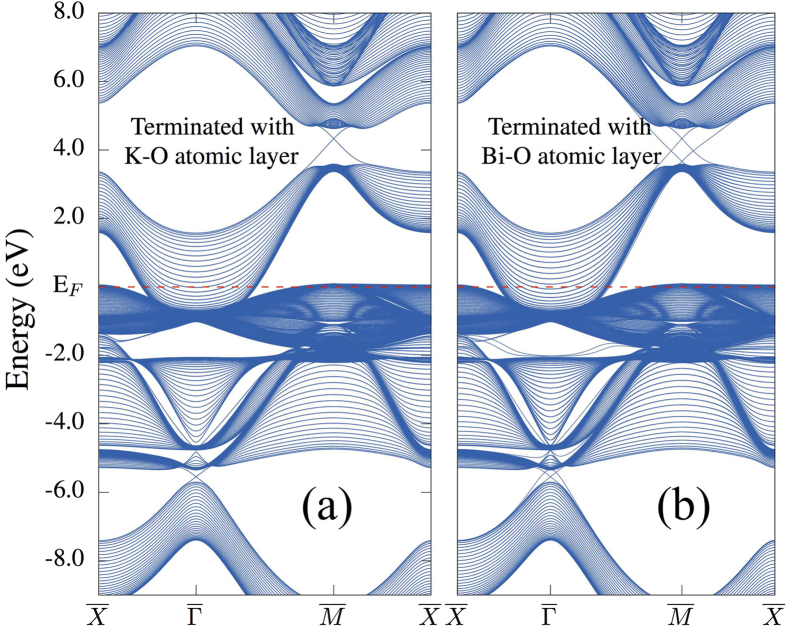
The surface states of cubic KBiO_3_ with two different types of surface terminations: (a) the upmost surface is K-O; (b) the upmost surface is Bi-O. In both setups, the bottom surface is taken as K-O. Both the top and bottom surfaces can hold Dirac surface states, and the corresponding Dirac cones are located at slightly different energies. The surface BZ is indicated by the green area in Fig. 1(c).

**Figure 3:**
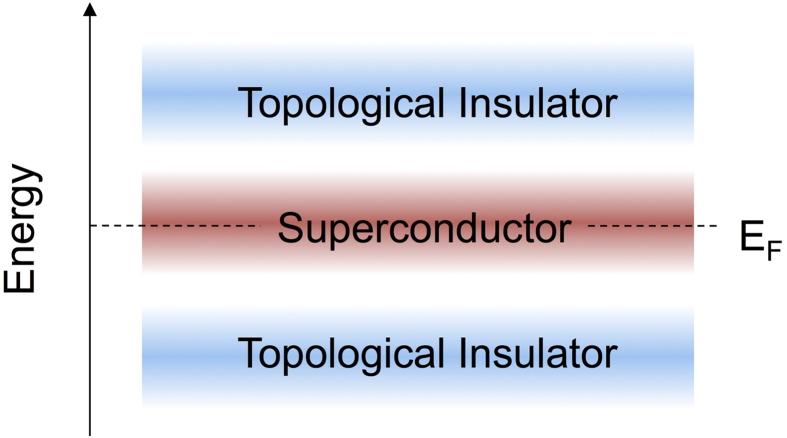
A schematic plot indicating the coexistence of the topological insulating and the superconducting phase versus energy in the Bismuth Oxide family.

To further validate the topological nature of cubic KBiO_3_ at both hole- and electron-doped regimes, we calculate the surface states by constructing a slab geometry along the [001] direction of bulk KBiO_3_. Fig. 2(a, b) displays the electronic structure of such a slab with thickness of 60 atomic layers. As one can clearly see from Fig. 2(a), there are two Dirac cones located at 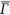 and 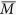. They stay at different sides of the Fermi level. Though, the energy gap below E_F_ is smaller than that at the R-point (above E_F_), it is clear that there is also a Dirac-cone positioned inside it. Thus, both topological surface states can, in principle, be accessed via “chemical engineering”, see the schematic plot in Fig. 3.

By calculating the projected density of states, we, further, verified that both Dirac cones are stemming from surface states (results are not shown here). With the current slab setup displayed in Fig. 2, each branch of the surface states is doubly degenerate as this slab contains two surfaces with the same atomic layer, which do not interact with each other. This degeneracy can be removed by terminating one surface with a different atomic layer, as shown in Fig. 2(b). When one surface is terminated by the Bi-O layer, while keeping the other one unchanged (K-O layer), the different potentials at these two surfaces separate the two Dirac cones in energy, which lifts the degeneracy. Furthermore, the Z_2_ topological invariant calculated in Table 1 also confirms KBiO_3_ to be a strong topological insulator.

**Figure 4:**
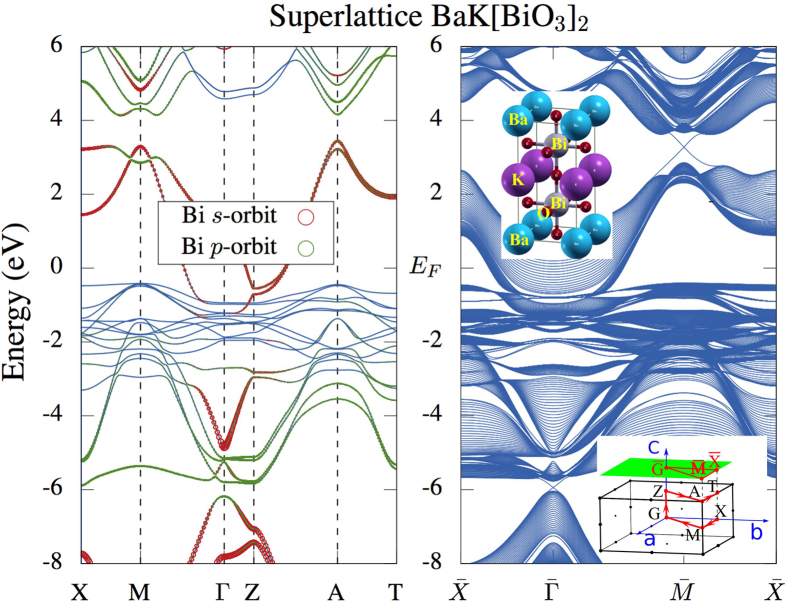
The bulk and surface electronic structures of a superstructure composed out of BaBiO_3_ and KBiO_3_.

Since both cubic BaBiO_3_ and KBiO_3_ can be TIs, we next show that their *random alloys in the same cubic phase*, in principle, can be also TI. Ba_1-x_K_x_BiO_3_ is found to crystallize in the Pm
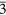
m phase for 0.3 < x < 0.5^13^. By taking the experimental lattice constant a = 4.2618 Å^13^ and setting c = 2a (relaxation on c does not change the following conclusion), we calculated the electronic structure of Ba_0.5_K_0.5_BiO_3_ by using a supercell with two Bi atoms (see Fig. 4 for the corresponding crystal structure and the BZ). Comparing with the cubic structure of BaBiO_3_ and KBiO_3_, the length of the c-axis is doubled. This leads to a folding of the BZ and to a mapping the R-point of the cubic BZ (see the BZ in Fig. 1) to the M-point. So does the band-inversion.

We found both band-inversions in cubic BaBiO_3_ and KBiO_3_ to exist in their corresponding superstructure. Now they are at the TRIM M-point and Γ-point. Here, we adopted the case x = 0.5 as an example to illustrate the surface states. This case allows us to work with a superlattice that is only twice the size of the cubic cell. As displayed in the right plot of Fig. 4, in a slab of Ba_0.5_K_0.5_BiO_3_ constructed with the superlattice bulk band structure along the [001] direction, these two band-inversions induce Dirac cones located at the 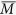 and 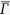 points at the surface BZ. Thus, substituting Ba with K does not change the topological nature of the system, see also Table 1 for the topological invariant. The band inversion, as well the Dirac cones are robust to this substitution, as long as the alloys crystallize in the cubic structure.

If we, further, compare the location of the energy gap at the R-point in three different systems, *i.e.* BaBiO_3_, KBiO_3_ and the superlattice BaK[BiO_3_]_2_, the potassium doping effect becomes obvious: K contains less valence electrons compared to Ba. Thus, substituting Ba with K effectively dopes holes into the system. As a result, the electronic structure of KBiO_3_ can be essentially understood as that of BaBiO_3_ with a global shift to a larger positive value in energy.

### BaPbO_3_ and BaPb_0.7_Bi_0.3_O_3_

The Bi *s-p* band inversions in BaBiO_3_ are left unaffected with the substitution of Ba with K, as the Ba and K states all stay at binding energies outside of the relevant energy regime for this band order reversal. Thus, it is not surprising that their alloys Ba_1-x_K_x_BiO_3_, in principle, also stay as TIs in the cubic phase. However, this is not the case in the BaPb_x_Bi_1-x_O_3_ systems. Chemically, Pb is adjacent to Bi in the periodic table, with also a large atomic number and, thus, a strong SOC (but weaker than that of Bi). A similar topological phase can then be expected.

From the following discussions, we will see that the relatively stronger SOC of Bi is crucial for realizing the topological phase in BaBiO_3_. By substituting Bi with Pb, the reduced SOC leads to a strong shift of the Pb *s*-type band, which, in the end, pushes the spin-filtered surface states into the bulk states.

**Figure 5:**
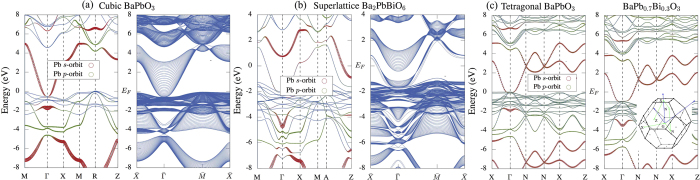
The electronic structure of BaPbO_3_ in the cubic (a) and tetragonal phases (c). The corresponding surface states in a [001] slab are presented in Figs. 5(a, b) in blue color. (**b**) shows the calculations for a superstructure of Ba_2_PbBiO_6_, which qualitatively displays the substitution effect of Bi with Pb in the random alloy BaPb_x_Bi_1-x_O_3_.

At temperatures above 673 K, BaPbO_3_ crystallizes in the same cubic structure as BaBiO_3_. Fig. 5(a) displays the electronic structures for the bulk and an [001] slab of cubic BaPbO_3_. The lattice constant is 4.2997 Å, taken from ref.^11^. Replacing Bi with Pb strongly modifies the band-inversions at R and Γ points. On the one hand, the band-inversion in cubic BaPbO_3_ moves to an energy of ~2 *eV* higher than that in BaBiO_3_. This is similar to what happens in KBiO_3_, *i.e.* Pb contains less valence electrons than Bi. Thus, replacing Bi with Pb effectively dopes the system with holes, which shifts the Fermi level downwards.

On the other hand, compared to BaBiO_3_ and KBiO_3_ (see Fig. 1), the details of the bulk electronic structure are also strongly modified. The *s*-type band moves to higher binding energy. At R around 5 *eV*, the *p*-type bands shows stronger dispersion along R-M and R-Z directions and the indirect energy gap here becomes negative. Thus, it is of no surprise to see that, when cubic BaPbO_3_ is terminated with a surface, the surface states originating from this band-inversion merge into the bulk states (see the blue-colored plot of Fig. 5(a)). With the tetragonal distortion (Fig. 5(c)), the band-inversion around 5 *eV* is still preserved (see also Table 1 for the topological invariant), which now appears at Γ. The separation of the Pb *s*-type and *p*-type bands in energy is also similar to that in the cubic phase (see Fig. 5(a)). For the same reason, the Dirac cone of the tetragonal BaPbO_3_ and BaPb_0.7_Bi_0.3_O_3_ also merge into the bulk bands but with their topological nature preserved.

In the right-hand plot of Fig. 5(c), the electronic structure of the tetragonal alloy BaPb_0.7_Bi_0.3_O_3_ is studied by using the virtual cluster approximation. *This is exactly the phase where superconductivity is observed in experiments.* The band inversion survives the tetragonal distortion and the chemical substitution. Thus, one may be tempted to speculate about topological superconductivity emerging from the coexistence of the superconductivity at T < T_c_ and the topological insulating nature. It is of crucial importance, however, to note that while the topological insulating phase can indeed be achieved on the basis of our electronic structure calculations, one is actually comparing two different systems (see also Fig. 3): one stands for the doped “high-T_c_” superconductor with the chemical potential E_F_ as given by our calculations. The other, *i.e.* the nominal TI system, requires an *additional* shift in the chemical potential to the energies, where the band inversion takes place. However, we find that, in both K- and Pb-doped BaBiO_3_, the topological gaps below the Fermi level are present. Particularly, in the Pb-doped BaBiO3, the Pb substitution of Bi displays clearly different influences on the topological gaps at above and below the Fermi level. The band-inversion and the energy gap *below* the Fermi level turns out to be more robust, which gives rise to surface states displayed in the right-hand plots in Fig. 5(a) and (b), respectively. If the two surfaces of the slab are terminated with different atomic-layers, one of the Dirac cones will merges into the bulk states, while the other one still is positioned inside the energy gap (see again the surface states plots in Fig. 5 (a) and (b)).

## Conclusions

In summary, it is a fascinating observation to explore topological band structure features “hidden” in the family of high-Tc superconductors, surrounding the BaBiO_3_ compounds. We found (1) the existence of an additional topological gap and the Dirac cone below the Fermi level, which was not discovered in the previous work^1^. This largely enriches the possibility of observing the Dirac cone in experiment. It should be possible to detect this lower Dirac cone in ARPES; (2) the topological nature of BaBiO_3_ is robust with respect to K- and Pb-doping. We believe our work represents one crucial step forward towards the final experimental realization of the topological phase of BaBiO_3_. The robustness of the topological nature of BaBiO_3_ can make the electric gating much easier in electron doped-BaBiO_3_ than in the parent compound.

The topological insulator properties and the corresponding Dirac cones embedded within a band gap appear at a different chemical potential than the superconducting state. However, they coexist in the same material class. The SC and the TI states may be related via chemical doping, or other means (see Fig. 3). Our results demonstrate in particular, that, apart from the chemical potential shift, the overall electronic structure remains essentially unchanged under doping, like in a “rigid band structure” description. This observation substantially enhances the possibility of switching from one unconventional state to the other. In that sense, our findings may open up new avenues for the above-mentioned possible applications, such as the fabrication of controlled SC/TI interfaces^31^.

## Methods

The calculations were mainly carried out within the full-potential linearized augmented plane-wave (FP-LAPW) method^18^, implemented in the package WIEN2k^19^. The package ELK (http://elk.sourceforge.net) and the Vienna Ab Initio Simulation Package (VASP)^20^,^21^,^22^,^23^ with PAW potentials^24^,^25^ are also employed for comparison. K_max_R_MT _= 9.0 and a 10 × 10 × 10 k-mesh were used for the ground-state calculations in WIEN2k. R_MT_ represents the smallest muffin-tin radius and K_max_ is the maximum size of reciprocal-lattice vectors. The spin-orbit coupling is included by a second variational procedure. The generalized gradient approximation (GGA) potential^26^,^27^ is used in all calculations. The Z_2_ invariant for all parent compounds are calculated within WIEN2k.

The surface electronic structures are further calculated using the maximally localized Wannier functions (MLWFs)^28^, employing the WIEN2WANNIER^29^ and VASP2WANNIER90^30^ interfaces. The MLWFs are constructed in a non-self-consistent calculation with an 8 × 8 × 8 k-mesh.

## Additional Information

**How to cite this article**: Li, G. *et al.* Topological nature and the multiple Dirac cones hidden in Bismuth high-Tc superconductors. *Sci. Rep.* **5**, 10435; doi: 10.1038/srep10435 (2015).

## Figures and Tables

**Table 1:** Topological invariants for all five compounds studied in this work. For all states below the topological gaps at above and below the Fermi level, the products of their parity eigenvalues are shown at all eight time-reversal invariant momenta. The Z_2_ topological invariant then verifies that they are all strong TIs.

	**For the topological gap above E**_**F**_	**Z**_**2**_	**For the topological gap below E**_**F**_	**Z**_**2**_
KBiO_3_ (Pm 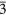 m)	1 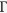 (+), 3X(+), 3M(+), 1R(−)	(1;111)	1 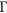 (-), 3X(-), 3M(-), 1R(+)	(1;111)
BaK[BiO_3_]_2_ (P4/mmm)	1 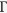 (+), 2X(+), 1M(−), 1Z(+), 1A(+), 2T(+)	(1;110)	1 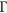 (−), 2X(+), 1M(+), 1Z(−), 1A(−), 2T(+)	(1;110)
BaPbO_3_ (Pm 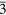 m)	1 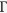 (+), 3X(+), 3M(+), 1R(−)	(1;111)	1 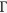 (−), 3X(−), 3M(−), 1R(+)	(1;111)
Ba_2_PbBiO_3_ (P4/mmm)	1 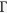 (+), 2X(+), 1M(−), 1Z(+), 1A(+), 2T(+)	(1;110)	1 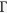 (+), 2X(+), 1M(−), 1Z(−), 1A(−), 2T(−)	(1;110)
BaPbO_3_ (I4/mcm)	1 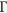 (−), 2X(+), 4N(+), 1Z(+)	(1;000)	1 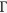 (+), 2X(−), 4N(+), 1Z(−)	(1;000)
